# Associations of neighborhood area level deprivation with the metabolic syndrome and inflammation among middle- and older- age adults

**DOI:** 10.1186/1471-2458-14-1319

**Published:** 2014-12-23

**Authors:** Akilah Dulin Keita, Suzanne E Judd, Virginia J Howard, April P Carson, Jamy D Ard, Jose R Fernandez

**Affiliations:** Institute for Community Health Promotion, Brown University, Box G-S121-8, Providence, USA; Department of Biostatistics, University of Alabama at Birmingham, 1530 3rd Avenue South, Birmingham, AL 35205 USA; Department of Epidemiology, University of Alabama at Birmingham, Ryals 210 F, 1530 3rd Avenue South, Birmingham, AL 35205 USA; Department of Epidemiology and Prevention, Wake Forest School of Medicine, Winston-Salem, USA; Department of Nutrition Sciences, University of Alabama at Birmingham, 1675 University Blvd., Webb 449A, Birmingham, AL 35205 USA

**Keywords:** Neighborhoods, Socioeconomic factors, Metabolic syndrome, Cardiovascular disease

## Abstract

**Background:**

The study examines the association of neighborhood socioeconomic deprivation and metabolic syndrome with inflammation.

**Methods:**

The analysis included 19, 079 black and white participants from the *RE*asons for *G*eographic *A*nd *R*acial *D*ifferences in *S*troke Study who were age > 45 years at baseline. Logistic regression examined whether neighborhood deprivation was associated with increased odds of METS and CRP-MetS.

**Results:**

Among black adults, residing in the most deprived neighborhoods was associated with increased odds of obesity (p < .01), lower HDL (p < .001), high blood pressure (p < .01), elevated fasting glucose (p < .001), inflammation (p < .01), and CRP-MetS (p < .001). Among white adults, neighborhood deprivation was associated with higher waist circumference (p < .001), lower HDL (p < .001), higher triglycerides (p < .01), higher glucose (p < .001), higher BMI (p < .0001), higher blood pressure (p = .01), METS (p < .001), inflammation (p < .01) and CRP-MetS (p < .001).

**Conclusions:**

These findings highlight the role of neighborhood socioeconomic deprivation on METS and CRP-MetS for black and white adults. Interventions tailored to address the contextual effects of deprived neighborhoods may reduce the observed neighborhood disparities.

**Electronic supplementary material:**

The online version of this article (doi:10.1186/1471-2458-14-1319) contains supplementary material, which is available to authorized users.

## Background

In the United States, cardiovascular disease (CVD) remains the leading cause of death and accounts for 16 percent of national health expenditures [1]. Medical costs of CVD have increased at an annual rate of 6 percent and it is projected that by 2030, 40 percent of the population will have some form of CVD [2]. Taken together, these statistics provide evidence of the social and economic consequences of CVD and suggest that CVD will significantly impact the quality of life for a large proportion of the American population. Due to these increased burdens, the identification of CVD predictors and reduction of risk factors are critical to reverse these expected trends. While researchers have devoted significant attention to the biological factors and health-related behaviors that contribute to CVD [3–5], there is a need for increased research focus on the relationship between social determinants, such as neighborhood socioeconomic deprivation, and CVD risk factors, particularly using clinically relevant biological markers of CVD risk.

While there are several plausible mechanisms through which neighborhood socioeconomic deprivation may increase CVD risk such as reduced access to health promoting behaviors, built environmental conditions that impede physical activity and exposure to environmental pollutants [6–8], contextual level socioeconomic status may also be an important pathway through which neighborhoods affect CVD. Individuals in lower socioeconomic status contexts report greater exposure to chronic social stressors, greater severity of stressors, and more daily hassles [9–13]. These SES-related stressors may contribute to biological wear and tear on the body leading to earlier health deterioration, or ‘weathering’, of individuals who reside in socioeconomically deprived neighborhoods [14]. Further, these differential stressor exposures may result in allostatic load which is the chronic overactivity or underactivity of allostatic systems (i.e. hypothalamic pituitary adrenal axis, the autonomic nervous system, insulin, immune and the metabolic systems-thyroid axis) [11]. During times of perceived stress, these systems are activated to protect the body [11]. Repeated activation of these systems may alter blood lipids, blood pressure and result in prolonged circulation of stress hormones and inflammatory cytokines that increase CVD risk [11, 15–17].

Previous studies report associations between neighborhood socioeconomic deprivation and CVD risk factors such as less participation in health promoting behaviors, increased exposure to psychosocial stressors and greater prevalence of obesity, type 2 diabetes and hypertension [6, 18–22]. Cumulatively, these relationships confer increased risks for CVD. The effects of place are further evidenced by the association of neighborhood socioeconomic deprivation with increased CVD among adults in international studies, in the United States and across racial/ethnic groups suggesting that neighborhood context has significant consequences for health [8, 18, 23–25]. While the aforementioned research is informative, the preponderance of evidence addresses health behaviors or single markers of CVD risk. Examining the clustering of factors that relate to CVD, such as the metabolic syndrome and its components may warrant attention. As the metabolic syndrome is an underlying factor for CVD, analyses of the relationships between neighborhood deprivation and metabolic syndrome factors including insulin resistance, impaired fasting glucose, hypertension, high triglycerides, low high-density lipoprotein (HDL), high waist circumference [26], and metabolic syndrome with an inflammation [27], may provide further insight into the mechanisms through which CVD differs by level of neighborhood deprivation. Research findings suggest that neighborhood deprivation is significantly associated with the metabolic syndrome, yet this remains a relatively understudied area particularly using population based data that includes a biracial cohort of black and white adults. Therefore, the objective of this research is to assess the associations of socioeconomic status on the metabolic syndrome components beyond individual level socioeconomic status and examine whether neighborhood socioeconomic deprivation is associated with biological markers of the metabolic syndrome among a population based cohort of middle- and older age black and white adults.

## Methods

The REasons for Geographic and Racial Differences in Stroke (REGARDS) study is a cohort of 30,239 black and white participants who, at the time of enrollment (January 2003-October 2007), were over age 45 and residing in the lower 48 states of the United States. The study was designed specifically to examine racial and regional differences in stroke mortality and therefore oversampled African Americans and those residing in the Southeast, a region commonly referred to as the stroke belt due to the high rates of stroke mortality in this region [28]. In REGARDS, the stroke belt is defined as Tennessee, Arkansas, Louisiana, Mississippi, Alabama, Georgia, South Carolina, and North Carolina. The study has been described elsewhere [29].

Study related protocol received approval from the Institutional Review Boards at all participating universities. University of Alabama at Birmingham, University of Vermont and State Agricultural College (Burlington), Wake Forest University School of Medicine (Winston-Salem, NC), Alabama Neurological Institute (Brookwood Medical Center), and University of Arkansas for Medical Sciences approved the study methods. REGARDS protocol included both a telephone based interview and an in-home visit. Individuals were identified from commercially available lists of residents, and recruited using an initial mailing followed by telephone contact. Using a computer-assisted telephone interview, trained interviewers obtained demographic information, medical history and indices of quality of life. Consent was obtained verbally and later in writing. Approximately three to four weeks after the telephone interview, trained personnel conducted a brief physical exam that included obtaining blood pressure measurements, blood samples, and an electrocardiogram. Phlebotomy was performed by centrally trained personnel using standardized procedures after a 10–12 hour fast. Within 2 hours of collection, samples were centrifuged and serum or plasma separated and shipped overnight in transfer vials on gel ice packs to the central laboratory at the University of Vermont. Overnight shipping was successful for 94% of participants with available samples. Samples were then re-centrifuged at 30,000 xG and 4 degrees Celsius, and either analyzed (general chemistries) or stored at -80 degrees Celsius.

For the current study, excluded participants included those whose addresses could not be matched at the block group level (n = 6413), who were not fasted (n = 2863), missing any component of the metabolic syndrome (n = 1870), and missing education (n = 14). The final analytic sample included 19,079 participants.

### Measures

Obesity, metabolic syndrome components and C-reactive protein. Obesity. Height was measured using a portable stadiometer without shoes and to the nearest 0.1 centimeter. Weight was measured using a digital scale. Body mass index (BMI) was calculated using the formula kg/m^2^. Waist circumference was measured mid-way between the lowest rib and the iliac crest in the standing position. Triglycerides and HDL-cholesterol were measured in serum using the Ortho Vitros Clinical Chemistry System 950IRC instrument (Johnson & Johnson Clinical Diagnostics, Rochester, NY)*,* which uses colorimetric reflectance spectrophotometry on thin film technology. The C.V.’s for HDL and triglyceride were 7% and <2%, respectively. During the in-home visit, systolic (SBP) and diastolic blood pressure (DBP) were measured twice with participants in the seated position. The average of the two measurements was calculated and used for analysis. Fasting glucose was measured in serum using a colorimetric reflectance spectrophotometry on the Ortho Vitros 950 IRC Clinical Analyzer (Johnson & Johnson Clinical Diagnostics, Rochester, NY) with a C.V. of 1%. C-reactive protein (CRP) was measured in SCAT-1 plasma using the BNII nephelometer from Dade Behring (Deerfield, IL) utilizing a validated high-sensitivity particle enhanced immunonepholometric assay. The assay range is 0.175 – 1100 mg/L. Intra-assay C.V.’s range from 2.3 – 4.4% and inter-assay C.V.’s range from 2.1 – 5.7% [30]. Participants self-reported being on lipid lowering, antihypertensive and antidiabetic medications.

Metabolic syndrome was defined using the modified ATP III definition [26] and included the following criteria:

1. Triglycerides ≥150 mg/dL
2. HDL Cholesterol for men <40 mg/dL and for women <50 mg/dL or any lipid lowering medication
3. Blood Pressure ≥130/85 mm Hg or antihypertensive medication use
4. Fasting glucose ≥100 mg/dL or antidiabetic medication use
5. Waist Circumference for men >102 cm (>40 in) and for women >88 cm (>35 in) (high waist circumference)

Prevalence of metabolic syndrome was defined as having three or more of these criteria. Also considered was an alternative definition of metabolic syndrome that has demonstrated associations in prior studies to include CRP as a marker of inflammation (CRP greater than 3 mg/dL) [27]. Even with the six components, we considered having three or more as the definition of metabolic syndrome plus inflammation.

Neighborhood deprivation. As we had address information available, we were able to geocode the home addresses of participants down to the US Census block group level. We used the summary index developed by Diez-Roux [19] as a measure of neighborhood deprivation. This measure is a compilation of U.S. Census derived indicators of neighborhood SES (median household income, percentage of households with interest, dividend or rental income, median value of housing units, percentage of persons 25 or over with complete high school, percentage persons 25 or over with complete college, and percentage persons in executive, managerial, or professional specialty occupations). First, we created individual Z-scores for each individual indicator and then summed the values across all variables to provide an index of neighborhood level deprivation. We then created quintiles of the Z-score for use in analysis.

Demographic, behavioral and history of cardiovascular disease events. Participants self-reported age, sex, race (African American and white), highest level of education completed, total annual household income, current cigarette smoking and history of cardiovascular disease. We categorized history of cardiovascular disease as any self-reported myocardial infarction or “heart attack”, stroke, coronary artery bypass surgery, coronary angioplasty or stenting, or evidence of myocardial infarction from electrocardiogram.

### Data analysis

Due to the hypothesis that neighborhood effects may vary by race-ethnicity [31], we conducted race-stratified analyses of neighborhood deprivation associations with the metabolic syndrome. We examined the association of neighborhood deprivation and metabolic syndrome using both linear and logistic regression. For the linear regression we examined the distribution of all components of metabolic syndrome and metabolic syndrome plus inflammation and transformed where necessary (results located online as Additional file 1: Table S1). We then standardized each variable by dividing by the standard deviation so we could compare across category. Since we were performing 16 different tests, we used a stricter p value for significance of 0.01. For logistic regression we dichotomized each variable of interest using the cut-point shown above and examined quintiles of neighborhood deprivation as the exposure. We also examined linear tests for trend across quintile. We conducted race-stratified analyses for neighborhood deprivation associations with metabolic syndrome as that was our primary analysis of interest. We also conducted Wald type 3 post hoc analyses to examine whether neighborhood associations with the metabolic syndrome components were significantly different between black and white adults.

## Results

**Figure 1:**
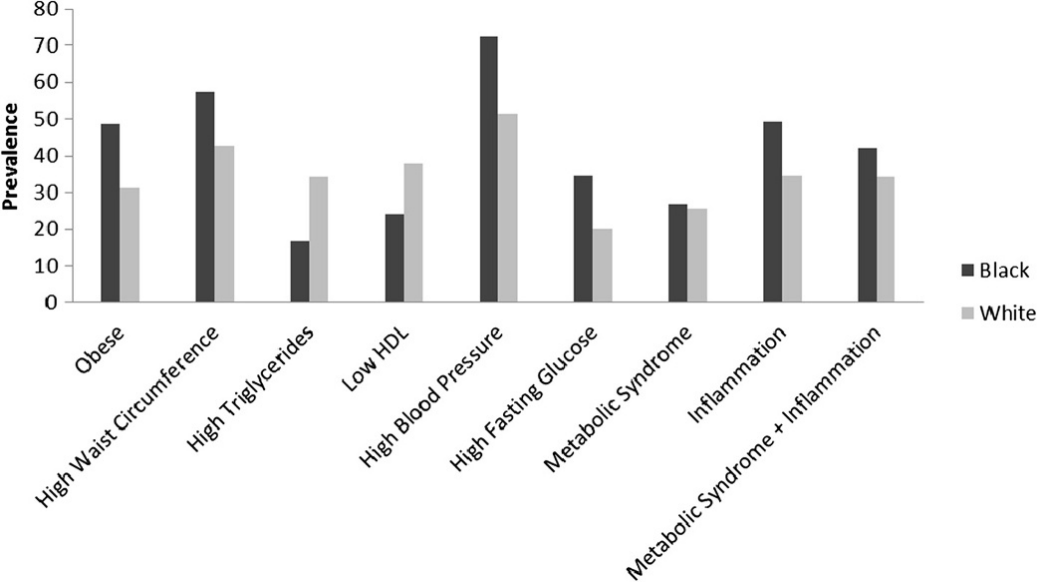
**Metabolic syndrome prevalence by race.**

Figure 1 presents the individual metabolic syndrome components, the prevalence of metabolic syndrome and metabolic syndrome with inflammation by race. Black adults had higher blood pressure, had higher waist circumference and were more obese. Similar percentages of black and white adults presented with the metabolic syndrome (26.7% and 25.5% respectively). A larger percentage of black adults had elevated C-reactive protein levels. When C-reactive protein was included in the metabolic syndrome criteria, a larger percentage of black adults presented with the metabolic syndrome (42.2% and 34.3% respectively).

Table 1 presents participant characteristics by quintile of neighborhood deprivation. There were statistically significant differences in age by neighborhood deprivation (p = 0.004) however, this translated into less than a one year age difference across neighborhood deprivation quintiles. A significantly greater percentage of black adults, females and individuals residing in the southeast lived in more deprived neighborhood conditions (p < .01). Neighborhood deprivation was significantly and positively associated with obesity, high waist circumference, high blood pressure, high fasting blood glucose, high triglycerides, low-HDL, the metabolic syndrome, and C-reactive protein (all significance levels p < .01). Neighborhood deprivation was also significantly associated with the metabolic syndrome with inflammation (p < .0001), and self-reported history of cardiovascular disease.

**Table 1:** **Descriptive statistics for the sample presented by quintile of neighborhood deprivation, higher quintiles indicate less neighborhood deprivation**

	N (Percentage)					
Characteristic	Quintile 1	Quintile 2	Quintile 3	Quintile 4	Quintile 5	p value
Median z score	-6.11	-3.21	-0.55	2.58	7.67	
Age (mean, SD)	64.5 ± 9.5	64.1 ± 9.3	64.4 ± 9.4	64.8 ± 9.4	64.9 ± 9.4	.004
Black	2706 (72)	1904 (51)	1397 (37)	1022 (27)	438 (12)	<.001
Female	2309 (62)	2191 (58)	2036 (54	1961 (52)	1838 (49)	<.001
Residing in Southeast	2631 (70)	2591 (69)	2404 (64)	2074 (55)	1928 (51)	<.001
^a^History of cardiovascular disease	898 (24)	832 (22)	790 (21)	723 (19)	698 (19)	<.001
Obese (BMI > =30)	1749 (47)	1642 (44)	1449 (39)	1292 (34)	1052 (28)	<.001
High waist circumference (>102 cm (>40 in) for men; > 88 cm (>35 in) for women	2116 (56)	2023 (54)	1875 (50)	1700 (45)	1396 (37)	<.001
High triglycerides (>150 mg/dL)	875 (23)	1025 (28)	1118 (30)	1066 (28)	1021 (27)	<.001
Low HDL cholesterol (for men <40 mg/dL, for women < 50 mg/dL or on lipid lowering medication)	1413 (38)	1433 (38)	1415 (38)	1354 (36)	1112 (30)	<.001
Blood pressure > 130/> 85 mm Hg or on antihypertensive medications	2574 (69)	2410 (64)	2210 (59)	2069 (55)	1724 (46)	<.001
Fasting glucose > 110 mg/dL or on antidiabetic medications	1070 (29)	912 (24)	772 (21)	585 (16)	444 (12)	<.001
Metabolic syndrome	992 (26)	958 (25)	911 (24)	785 (21)	598 (16)	<.001
Inflammation (CRP ≥ 3 mg/L)	1853 (49)	1701 (45)	1505 (40)	1392 (37)	1115 (30)	<.001
Metabolic syndrome + inflammation	1722 (24)	1615 (23)	1482 (21)	1323 (19)	1003 (14)	<.0001
Current smoking	751 (20)	676 (18)	534 (14)	445 (12)	316 (8.5)	<.001
Income						
less than $20 K	1281 (34)	874 (23)	579 (16)	344 (9.2)	145 (3.9)	
$20 K–$34 K	1079 (29)	1064 (28)	1001 (27)	853 (23)	514 (14)	
$35 K–74 K	710 (19)	1039 (28)	1224 (33)	1422 (38)	1317 (35)	
$75+	171 (4.6)	340 (9.1)	487 (13)	722 (19)	1337 (36)	
Refused to report	510 (14)	451 (12)	455 (12)	417 (11)	443 (12)	<.001
Education						
<High school	946 (25)	597 (16)	374 (10)	189 (5.0)	79 (2.1)	
High school	1287 (34)	1192 (32)	1061 (29)	826 (22)	489 (13)	
Some college	869 (23)	1073 (28)	1132 (30)	1140 (30)	900 (24)	
College	649 (17)	906 (24)	1179 (31)	1603 (43)	2288 (61)	<.001

^a^History of cardiovascular disease defined as self-reported myocardial infarction (MI) or “heart attack”, stroke, coronary artery.bypass surgery, coronary angioplasty or stenting, or evidence of MI from electrocardiogram.

^b^Percentages may not add up to 100 due to rounding error.

Table 2 presents race specific odds ratios and 95% CIs of the metabolic syndrome and individual metabolic syndrome components. The results are presented by quintile of neighborhood deprivation with individuals in the least deprived neighborhoods (quintile 5), as the referent group.

**Table 2:** **Logistic regression models examining associations of quintile of neighborhood deprivation with the odds of metabolic syndrome components and inflammation (higher quintiles indicate less deprivation)**

Variable	Quintile 1	Quintile 2	Quintile 3	Quintile 4	Quintile 5
**Black Adults**					
Obese (BMI > =30)	1.27 (1.02,1.58)	1.25 (1.01,1.57)	1.21 (0.96,1.51)	1.07 (0.85,1.35)	Reference
High waist circumference (>102 cm (>40 in) for men; > 88 cm (>35 in) for women	1.23 (0.98,1.55)	1.25 (0.99,1.57)	1.25 (0.99,1.58)	1.05 (0.83,1.33)	Reference
High triglycerides (>150 mg/dL)	1.30 (0.95,1.77)	1.27 (0.93,1.74)	1.28 (0.93, 1.75)	1.06 (0.76,1.48)	Reference
Low HDL (for men <40 mg/dL, for women < 50 mg/dL or on lipid lowering medication)	1.38 (1.09,1.76)	1.27 (1.00,1.61)	1.28 (1.00,1.63)	1.07 (0.83,1.38)	Reference
Blood pressure > 130/> 85 mm Hg or on antihypertensive medications	1.34 (1.07,1.68)	1.32 (1.05,1.66)	1.20 (0.95,1.51)	1.09 (0.86,1.38)	Reference
High fasting glucose > 110 mg/dL or on antidiabetic medications	1.41 (1.10,1.79)	1.41 (1.11,1.80)	1.29 (1.01,1.65)	1.12 (0.87,1.45)	Reference
Metabolic syndrome	1.19 (0.91,1.57)	1.19 (0.90,1.57)	1.18 (0.89,1.57)	0.96 (0.71,1.29)	Reference
Inflammation (CRP ≥ 3 mg/L)	1.36 (1.09,1.70)	1.23 (0.98,1.54)	1.21 (0.97,1.52)	1.17 (0.93,1.48)	Reference
Metabolic syndrome with inflammation	1.52 (1.20,1.92)	1.39 (1.10,1.77)	1.36 (1.07,1.74)	1.13 (0.88,1.45)	Reference
**White Adults**					
Obese (BMI > =30)	1.69 (1.44,1.98)	1.69 (1.48,1.92)	1.40 (1.24,1.58)	1.21 (1.08,1.36)	Reference
High waist circumference (>102 cm (>40 in) for men; > 88 cm (>35 in) for women	1.58 (1.36,1.84)	1.59 (1.40,1.80)	1.44 (1.29,1.61)	1.29 (1.16,1.44)	Reference
High triglycerides (>150 mg/dL)	1.20 (1.03,1.41)	1.20 (1.05,1.36)	1.27 (1.13,1.42)	1.13 (1.01,1.27)	Reference
Low HDL (for men <40 mg/dL, for women < 50 mg/dL or on lipid lowering medication)	1.44 (1.23,1.67)	1.46 (1.28,1.67)	1.41 (1.26,1.59)	1.37 (1.23,1.53)	Reference
Blood pressure > 130/> 85 mm Hg or on antihypertensive medications	1.13 (0.97,1.31)	1.20 (1.06,1.36)	1.14 (1.02,1.28)	1.14 (1.03,1.26)	Reference
High fasting glucose > 110 mg/dL or on antidiabetic medications	1.58 (1.32,1.89)	1.43 (1.23,1.67)	1.32 (1.15,1.52)	1.07 (0.93,1.22)	Reference
Metabolic syndrome	1.78 (1.50,2.12)	1.62 (1.39,1.88)	1.56 (1.36,1.79)	1.38 (1.21,1.57)	Reference
Inflammation (CRP≥3 mg/L)	1.25 (1.07,1.45)	1.37 (1.21,1.56)	1.19 (1.06,1.35)	1.17 (1.05,1.31)	Reference
Metabolic syndrome with inflammation	1.65 (1.41,1.93)	1.67 (1.46,1.91)	1.55 (1.37,1.75)	1.37 (1.22,1.54)	Reference

Model adjusted for age, sex, region, smoking, individual level education and income.

N = 18,779.

Relative to black adults living in the lowest level of deprivation, black adults residing in the highest level of deprivation had 21% increased odds of obesity (BMI ≥ 30 kg/m^2^) (OR = 1.27, 95% CI 1.02 to 1.58). Relative to the lowest level of neighborhood deprivation, black adults residing in the highest level of deprivation had 38% higher odds of low HDL (OR = 1.38, 95% CI = 1.09 to 1.61). Black adults in the highest level of deprivation had a 34% increased odds of high blood pressure (OR = 1.34, 95% CI 1.07 to 1.68). Living in the most deprived neighborhood was associated with a 41% increased odds of elevated fasting glucose (OR = 1.41, 95% CI = 1.10 to 1.79). Relative to the least deprived, black adults residing in the highest level of deprivation had 36% increased odds of inflammation (OR = 1.36, 95% CI = 1.09 to 1.70). When inflammation was added to the metabolic syndrome criteria, black adults residing in the highest level of deprivation had 52% higher odds of the metabolic syndrome (OR = 1.52, 95% CI 1.20 to 1.92).

White adults in quintile 1 had a 69% increased odds of obesity relative to white adults living in the least deprived neighborhoods (quintile 1OR = 1.69, 95% CI = 1.36 to 1.84). Residing in the most deprived neighborhood was associated with 58% increased odds of high waist circumference (quintile 1 OR = 1.58, 95% CI = 1.36 to 1.84). White adults residing in the lowest level of deprivation had a 20% increased odds of elevated triglycerides (OR = 1.20, 95% CI = 1.03 to 1.41). Compared to white adults living in the lowest level of deprivation, white adults in the most deprived neighborhoods had 44% increased odds of low HDL (OR = 1.44, 95% CI = 1.23 to 1.67). For white adults residing in the highest level of deprivation, there was a 58% increased odds of elevated fasting glucose (OR = 1.58, 95% CI = 1.32 to 1.89). Relative to individuals in the least deprived neighborhoods, white adults residing in the highest level of neighborhood deprivation had 25% increased odds of inflammation (OR = 1.25, 95% CI = 1.07 to 1.45). Among white adults, residing in the most deprived neighborhood was associated with 78% increased odds of the metabolic syndrome (OR = 1.78, 95% CI = 1.50 to 2.12). When inflammation was added to the metabolic syndrome, residing in the most deprived neighborhood was associated with 65% increased odds of metabolic syndrome (OR = 1.65, 95% CI = 1.41 to 1.93). Post hoc analyses did not indicate any statistically significant differences in the relative odds of metabolic syndrome components across neighborhood quintiles between black and white adults (data not shown).

## Discussion

The current study examines the relationship of area level deprivation and its associations with the metabolic syndrome and inflammation among a national sample of black and white middle- and older age adults. Given the stringent criteria to establish significance and the availability of objective measures of the metabolic syndrome, the study findings provide strong evidence that neighborhood deprivation is associated with the metabolic syndrome. These findings may be lend support to a stressor and allostatic load framework as potential mechanisms through which neighborhood deprivation is associated with the metabolic syndrome. In the race-stratified analyses, independent neighborhood associations persisted after accounting for individual-level socioeconomic status, age, sex, health behaviors and geographic region.

The study findings indicate that living in more deprived neighborhoods is associated with increased odds of the individual metabolic syndrome components for both white and black middle- and older age adults. The findings are also consistent with the research findings among participants in the Canadian Cohort Study in that neighborhood deprivation is associated with increased odds of hypertension [32]. Although the neighborhood economic indicators differ somewhat across studies, the current study findings are also similar to those of Bird et al., [18] in that neighborhood deprivation is associated with higher blood pressure and low-HDL cholesterol. The significant relationship of neighborhood deprivation on inflammation is also similar to findings from both MESA and Jackson Heart Study participants which indicate that neighborhood deprivation operates to affect health through inflammatory pathways [8, 33]. Neighborhood deprivation also confers statistically significant increases in the risk of impaired fasting glucose. This contrasts with the research of Andersen et al., [34] that suggests non-significant relationships between neighborhood deprivation and fasting glucose. However, that study did not include a national sample of adults and was limited to women. The significant associations evidenced among the REGARDS population align with the general literature suggesting neighborhood associations with insulin and glucose outcomes among MESA participants [8] and insulin resistance [35].

While neighborhood deprivation is associated with many of the metabolic syndrome components, the relationships differ for the anthropometric measures BMI and waist circumference. Among white adults, there are clear and significant relationships for high BMI and large waist circumference for each quintile of neighborhood deprivation compared to the least deprived neighborhoods. In contrast, the relationship of waist circumference is insignificant for black adults and the relationship of BMI is only significant for the two highest quintiles of deprivation relative to the least deprived neighborhoods. Research suggests that relationships between neighborhood context and obesity tend to be stronger for women than men and it is possible that if we conducted race-gender specific analyses, then it is possible that neighborhood associations with obesity would be observed among the black participants in REGARDS [22, 33, 36, 37]. However, nationally representative data from the ARIC study suggest that socioeconomic status is not significantly associated with large waist circumference among black participants and white men participants [36]. Among participants in the Baltimore Memory Study, there are weak or non-significant associations between neighborhood deprivation and the odds of obesity for both black and white older adults [38]. This suggests that the relationship between neighborhood deprivation and total body mass are complex and the nature of the relationships may differ across race, gender, and age categories.

Although differences in the odds of the metabolic syndrome are statistically significant for the white participants only, the differences in the odds of the metabolic syndrome with inflammation between the most and least deprived neighborhoods are large for both white and black participants. These findings differ from those of Merkin et al., [39] who identified that differences in metabolic and cardiovascular allostatic load scores between the most deprived relative to the least deprived neighborhoods are largest for black participants and non-significant for their white and Mexican American counterparts. However, the current study results are similar to those of Bird et al., [18] that there is no evidence of race/ethnic specific patterns in the association of neighborhood deprivation on the risk for high cardiovascular and metabolic allostatic load scores. While there are differences in measurement between the current study and the comparison studies, both suggest the significance of neighborhood deprivation on risk factors for CVD among nationally representative samples of black and white adults. Further, the current study suggests that the inclusion of inflammation in the metabolic syndrome has significant consequences for the black participants and may warrant inclusion in the traditional risk factor panel for metabolic syndrome criteria for this population. Aside from race-specific measures that may be needed to better define metabolic syndrome, conclusions about the relationship between neighborhood deprivation and CVD risk may also be affected by other physiologic mediators of metabolic syndrome that differ by race. For example, Onat et al. have suggested that Lp(a) mediates the incidence of metabolic syndrome via a U-shaped relationship, and non-blacks may be more likely to be in lower tertiles of Lp(a) with more atherogenic lipid profiles [40, 41]. While this is speculative, additional work to explore these hypotheses is necessary to determine if different pathways are at play in the expression of CVD risk that results from being exposed to deprived environments in various race/ethnic groups.

### Limitations

The current study has a number of strengths, including a large national sample of black and white middle to older age adults and the use of objective measurements of the metabolic syndrome. However, there are some limitations, the large proportion of those with missing data, particularly those who could not be geo-coded at the block group level may limit the current findings and the use of cross-sectional data limits the ability to infer causality. We are unable to establish whether individuals with poorer health are concentrated in less economically advantaged neighborhoods as a result of early childhood health problems that limit social mobility (health selection hypothesis) [42]. Longitudinal studies that control for the independent effects of childhood health and lifetime socioeconomic status are warranted to establish the temporal relationship and strength of the effects of neighborhood deprivation on the metabolic syndrome. Further, the Department of Housing and Urban Development sponsored research conducted by Ludwig and colleagues [43] provides strong evidence that neighborhood factors exert direct and causal effects in the development of metabolic syndrome risk factors. Additionally, the US Census boundaries to denote neighborhood deprivation at the block-group level, while the smallest unit of analysis available, may not correspond with individual participant definitions of neighborhood contexts. However, the Census derived measurements provide standardized neighborhood boundaries that allow for meaningful comparisons across research studies.

## Conclusions

The current findings contribute to the growing body of literature suggesting that there are independent neighborhood associations with the clustering of metabolic syndrome components. Future studies should simultaneously examine the multiple pathways (e.g. built, access to resources, environmental exposures, and social stressors) through which neighborhood context affects CVD risk to identify the relative importance of each and to develop meaningful interventions that might reduce the effects of neighborhood context on increased CVD risk.

## Electronic supplementary material

Additional file 1: Table S1: Standardized regression coefficients for the individual metabolic syndrome components and C-reactive protein. (DOC 40 KB)

## Abbreviations

METS: Metabolic syndrome
CRP-MetS: Metabolic syndrome and inflammation
CVD: Cardiovascular disease
HDL: High-density lipoprotein
BMI: Body mass index
SBP: Systolic blood pressure
DBP: Diastolic blood pressure
CRP: C-reactive protein.
