# Supplementary material for: Associations of neighborhood area level deprivation with the metabolic syndrome and inflammation among middle- and older- age adults
Source: BMC Public Health. 2014 Dec 23;14:1319. doi: 10.1186/1471-2458-14-1319 (PMC4364504; doi:10.1186/1471-2458-14-1319)
Supplement: Supplementary file 1 — Additional file 1: Table S1: Standardized regression coefficients for the individual metabolic syndrome components and C-reactive protein. (DOC 40 KB) [file 12889_2014_7454_MOESM1_ESM.doc]

**Additional file 1: Table 1** Standardized regression coefficients for the individual metabolic syndrome components and C-reactive protein

| **Variable** | **Model 1: Unadjusted** | **Model 2: Demographic and Behavioral^a^** | **Model 3: Demographic, Behavioral and socioecomonic^b^** |
| --- | --- | --- | --- |
| **Black Adults** |  |  |  |
| Obese (BMI>=30) | -0.076*** | -0.086*** | -0.055*** |
| High waist circumference (> 102 cm (> 40 in) for men; > 88 cm (> 35 in) for women | -0.049*** | -0.077*** | -0.040*** |
| High triglycerides (> 150 mg/dL) | -0.060*** | -0.059*** | -0.037*** |
| Low HDL (for men <40 mg/dL, for women < 50 mg/dL or on lipid lowering medication) | 0.042*** | 0.055*** | 0.055*** |
| Systolic blood pressure > 130 mm Hg or on antihypertensive medications | -0.118*** | -0.121*** | -0.074*** |
| Diastolic blood pressure >85 mm Hg or on antihypertensive medications | -0.031^**^ | -0.052*** | -0.026^**^ |
| High fasting glucose > 110 mg/dL or on antidiabetic medications | -0.077*** | -0.082*** | -0.038^**^ |
| Inflammation (CRP> 3 mg/L) | -0.124*** | -0.100 | -0.070*** |
| **White Adults** |  |  |  |
| Obese (BMI>=30) | -0.106*** | -0.126*** | -0.089*** |
| High waist circumference (> 102 cm (> 40 in) for men; > 88 cm (> 35 in) for women | -0.100*** | -0.128*** | -0.089*** |
| High triglycerides (> 150 mg/dL) | -0.117*** | -0.106*** | -0.050*** |
| Low HDL (for men <40 mg/dL, for women < 50 mg/dL or on lipid lowering medication) | 0.124*** | 0.132*** | 0.086*** |
| Systolic blood pressure > 130 mm Hg or on antihypertensive medications | -0.098*** | -0.100*** | -0.069*** |
| Diastolic blood pressure >85 mm Hg or on antihypertensive medications | -0.048*** | -0.054*** | -0.040*** |
| High fasting glucose > 110 mg/dL or on antidiabetic medications | -0.070*** | -0.066*** | -0.034*** |
| Inflammation (CRP> 3 mg/L) | -0.125*** | -0.107*** | -0.065*** |

^**^p>0.01, ***p≤0.001

a. Model 1 includes age, sex, region, smoking

b. Model 2 adds income and education to model 1
